# Case of Fissure of Sternum Permitting Examination of the Heart

**Published:** 1858-08

**Authors:** 


ART. VII.— *Case of Fissure of Sternum permitting examination of the Heart.* By Prof. HAMERNIK. Translated from the Wiener Medizinische Wochenschrift, (No. 29 to 32), by J. DA COSTA, M. D.

**Editor of Buffalo Medical Journal.**

I take the liberty of placing in your hands, for insertion in your journal, the accompanying translation which, my friend, Dr. Da Costa, has obligingly made and sent to me, not for publication, but on account of its interest and importance in connection with the studies to which my attention is at present directed. I have presumed upon his well known zeal in behalf of medical science, in assuming the responsibility of handing it to you for publication without his knowledge. In doing so, it is proper that a portion of his letter accompanying the translation should be prefixed.

Yours truly, A. F.

PHILADELPHIA, July 3d, 1858.

MY DEAR DR.:

I send you the long promised paper on a case of malformation of the sternum, which permitted the heart's action to be studied. It is certainly of interest, with reference to the much disputed point of the impulse of the heart.

The translation I send you is not entirely literal; the style of the author, more diffuse than even ordinary German writers, made this impossible. Here and there, I have left out statements and speculations, which had no very marked bearing on the phenomena in question. I cannot lay claim either, to my translation being well expressed, but if it answers your purpose, I shall be satisfied. Should you wish for any further information regarding the case, or rather some of the anatomical statements which are made in connection with it, please let me know; I have marked the passages where the author has introduced them.

Believe me,

Truly and respectfully yours,

J. DA COSTA.

Gronx, Alex.; age 23; merchant; in good health, although delicate; the malformation was observed from his birth.

At each inspiration, the fossa supra-clavicularis deepens a little, and the lower portion of the neck is drawn inwards; at the origin of the sterno-cleido mastoid a moderate sinking in occurs, which disappears again on expiration.

No venous murmurs in the neck.

The thorax has the usual form, but its diameters are proportionately smaller.

At the lower portion of the chest the measure to the vertebral column, (2d lumbar vertebra) shows on the right side 34, on the left 33 centim.; measured across the nipples on the right side 36½, left 38 cm.; under the axilla, right side 37, left 39½ cm.; greater prominence, evidently, also on inspection, between the 4th and 7th ribs near their insertion on the left side. The left nipple is 10½ cm. from the median line, the right 9. * * *

* * * Along the whole length of the sternum at the median line, there exists a fissure, which separates the bone into two portions, a right and a left half. This fissure becomes pointed and very narrow towards the ensiform cartilage, which keeps the two halves tolerably firm in position. The width of the fissure during quiet breathing, is:

Between the clavicles, 3 cm.

Between the 3d costal cartilage, 3½ cm.

Between the 6th and 7th cartilage, scarcely ½ cm.

Expiration diminishes the width of the fissure; a full, forced inspiration increases it, especially between the 3d and 4th ribs; it reaches then, 5 cm. During quiet breathing, the described fissure appears concave; this concavity is increased the stronger the inspiration.

During forced expiration the thorax and the abdomen becomes narrower; the veins of the neck become clearly discernible; the fissure becomes convex, is forced outwards, is several lines above the level of the skin, and has the appearance of a tumor, of oblong shape, pointed at one extremity. The fissure is covered only by skin and, perhaps, the thoracic fasciæ; this covering is very movable and yielding, permitting the respiratory and circulatory phenomena to be clearly witnessed.

The parietes of the front of the chest are very elastic; the cartilages of the ribs, especially of the 3d and 6th, and the corresponding portions of the sternum may thus be bent very considerably inwards, without occasioning, however, any abnormal phenomena or an alteration in the pulsations of the arteries of the upper or lower extremities.

The percussion sound at the thorax, is sufficiently "long" and continuous, (*i. e.*, during an expiration,) and *non-tympanitic.* [Long means, with the German writers, clear or sonorous; short, dull.]

On the right side of the chest the percussion sound becomes "short" near the sternum at the upper portion of the 4th rib, and in the axilla at the lower portion of the 7th rib; the dullness of the liver does not extend quite as far downwards below the rib, as is usual.

On the left side, between the 3d and 4th ribs, the percussion sound becomes shorter, at the upper part of the 4th rib quite "short," whilst there also the resistance to the percussing finger is correspondingly increased. At the 6th rib the sound becomes "longer," and at the lower portion of this rib, is of the usual length.

Between the 4th and 5th ribs the percussion sound is like that of the thigh, also equally dull at the adjoining portion of the left half of the sternum, and does not in the least change during deep respiration.

From the margin of the portion of the sternum just mentioned, the dullness measures outwards, at the 4th rib 6, at the 5th rib 8½, at the 6th rib 9 cm.; where the left lobe of the liver joins the 6th costal cartilage, the dullness measures from the inner margin of the left sternal half outwards, but it is not possible to determine whether the dull sound is produced by the heart or liver. The upper portion of the lung yield less clear sounds than usual.

The murmurs of respiration are not distinctly marked; at the posterior upper portion of the chest, sibilant sounds may sometimes be perceived, and in expiration a prolonged, rough sound.

The impulse of the heart is rather indistinct between the 5th and 6th ribs; it may be felt from the inner part of the left sternal half outwards, and, at one spot, in the intercostal space just indicated, a point exists, which can be covered with the tips of the fingers, and which during each ventricular systole, is raised and becomes slightly convex and hard, Between the 4th and 5th ribs, exactly at the nipple, a similar point is met with; at the 4th and 5th intercostal space, indistinct movements are felt, and near the nipple, may also be seen.

The sounds of the heart are all normal, except the diastolic sound at the pulmonary artery (2d and 3d ribs, left side) which is markedly increased.

All the arteries are delicate, and their pulsations not very distinct; it is only at parts of the chest and in the neck that the pulsations can be heard; at other arteries they are only felt.

72 contractions of the heart in a minute. Over the fissure the percussion sound, even by forced breathing, is not tympanitic, but like that on the right side of the chest. If the fissure situated between the two halves of the sternum be closely studied whilst the patient is breathing quietly, the following phenomena are witnessed:

At its uppermost part on a level with the 1st rib, the finger may *feel* the sounds of the arch of the aorta, which passes here past the fissure, *i. e.*, the known "tic-tak," or pulsation of the aorta, and the sound of its valves. Further downwards, changes in form, and in resistance can only be felt and seen occasionally, and then only during the systole of the heart. At other times the fissure remains to the touch, soft and yielding, and no changes can be perceived, even if the touching finger is strongly pressed inwards. Shortly before the impulse of the heart is felt (between the 5th and 6th ribs) there appears discernible to the eye and to the finger at the fissure, from the 2d rib downwards, a roundish body of moderate hardness, and producing a movement of the parietes. This body soon diminishes in all its diameters, especially from above downwards and from right to left, and when in its retraction, it arrives from the 2d right costal cartilage to the 4th costal cartilage of the left side, the impulse of the heart in this region becomes perceptible. This roundish body then disappears, at least to the touch, whilst a slight prominence of the soft parts becomes gradually visible, in the direction of above downwards and from the left to the right; then occurs again, the already described retraction and hardening, producing that roundish body only felt at this time. (It is the systole of the muscular fibres of the heart commencing in the right auricle, and extending to the ventricle.) It can be clearly seen, how this body, becoming at times by its hardness and its movement distinctly perceptible, (it is the right auricle,) still retains after this contraction a considerable extension, and only becomes inappreciable to the touch, because the hardening and the movements of its walls disappear.

During a forced inspiration, the fissure enlarges, loses its concavity, and the just mentioned movements appear less distinct. During a forced expiration, the undulations described becomes also less perceptible, as the skin covering the fissure is forced outwards in a convex form.

A close inquiry proves beyond doubt, that the movements perceived at the fissure are dependant upon a hardening and the vibrations of a spherical body, which diminishes in its diameters, but presents at the end of its contractions a not inconsiderable volume; it also proves that these movements commence before the impulse of the heart is felt, and end with them.

Here the description of the case terminates, but Dr. H. makes some remarks on it, of which the following are the most important: Considering the anatomy of the parts in normal persons, there is reason to believe that there is here no difference, that the two laminæ mediastini are attached to the left half of the sternum, that the portion of the circulatory apparatus lying in the fissure is covered by the edge of the right lung, and that this occasions the clear percussion sound, also that the right costal pleura extends across the fissure to the left sternal half and becomes continuous with the laminæ mediastini.

The fissure would then, in this case be immediately over the right pleura, portions of which and of the right lung would be stretched across the parts of the circulatory apparatus which lie under the sternum, (that is the right ventricle, auricle, the transverse portion of the arch of the aorta, and the ven. anonym. sinistra.)

[Dr. H. cites anatomical and pathological observations to prove the relative positions of the right lung and pleura and heart just mentioned.]

On a level with the first rib the arch of the aorta crosses the sternum, or in this case, the fissure. A finger there introduced finds the pulsation of the coats of the aorta, (systolic sound) and the sound of its closing valves (diastolic sound.)

In the short interval between the two sounds, nothing can be seen or felt at this portion of the fissure.

In the so-called horizontal position of the heart, its convex surface lies against the cartilages of the ribs. During the diastole the heart is larger than during its shortening, or systole, and touches, therefore, more of the thoracic walls. Now the fact that it is not perceived during the diastole, has given rise to the erroneous supposition that it recedes from the parietes of the chest during this act, and again, that by its coming up to the walls it occasions the heart's impulse during the systole. But it is not necessary to suppose that it recedes; it can simply not be perceived, because we cannot distinguish any muscle from the surrounding textures, unless that muscle be in a state of contraction, as in the case of cramps of the muscles of the leg.

The *contraction* thus of the muscles of the heart cause the organ to be felt. These contractions or vibrations are transmitted to the coats of the arteries, and occasions their pulsation.

The body which is distinctly perceived at the fissure between the 2d and 4th ribs, must be the right auricle; it is not felt during the diastole, because no muscles when not in a state of contraction can be felt as distinct bodies. The tolerable clear percussion at the fissure is owing to the margin of the right lung.

If the hand be placed over the seat of the impulse of the heart, it may here be seen how the shortening and hardening of the heart (systole) commences at the uppermost part of the right ventricle, about the 2d rib, and spreads downwards and to the left, until at the 4th rib, and at the left portion of the sternum the finger feels the beat of the heart and pulsation of the aorta.

The "impulse" of the heart is, therefore, the distinct perception of the contraction or systole, and is noticed at an intercostal space, against which the convex walls of the heart permanently lies. The systole begins in the auricles and, this case proves, spreads to the ventricles.

During the rest of the muscles of the heart, they could be perceived in this patient gradually to extend in the direction from the ventricles to the right auricle, then again commenced the systole.

During the systole, Dr. H. does not believe that the slightest twisting or change in position, either upwards or downwards takes place. The ventricles, like the auricles, alter their form; to suit this, the yielding part in front gives way, and we have the vibration best felt in the 5th intercostal space.

The surrounding diaphragm and liver, as well as the increase in the diameter of the thickness of the heart cause the vibrations to be best perceived there, because room is most easily made there for the encroaching organ. It cannot be downwards, as the adjacent liver puts stronger obstacles in the way.

The great dullness between the 4th and 5th ribs, not changed by forced respiration, is owing, probably, says Dr. H., to extensive and unyielding adhesions of the left pleura. The patient had suffered from fugitive pains in this region, and other symptoms which lead to this belief.

